# Two-stage surgery for intraperitoneal and retroperitoneal multicentric liposarcoma causing hydronephrosis: a case report

**DOI:** 10.1186/s40792-019-0576-y

**Published:** 2019-02-04

**Authors:** Ryohei Murata, Tadashi Yoshida, Nobuhiro Kobayashi, Yoshito Watanabe, Shigenori Homma, Hayato Echizenya, Akinobu Taketomi

**Affiliations:** 1 Department of Surgery, Otaru General Hospital, 047-8550, 1-1-1, Wakamatsu, Otaru-shi, Hokkai-do Japan; 2 Department of Gastroenterological Surgery I, Hokkaido University Hospital, 060-8648, Nishi 5 chome, Kita 14 jyo, Kita-ku, Sapporo-shi, Japan

**Keywords:** Intra-abdominal, Liposarcoma, Multicentric, Myxoid, Round cell, Retroperitoneal

## Abstract

**Background:**

Liposarcoma is a soft tissue sarcoma of adipocyte origin. Liposarcoma represents 20–30% of adult soft tissue tumors, which was most frequently seen in the retroperitoneal space in 45% and abdominal space in only 5% of cases, but the multicentric case is unknown. Herein, we describe a rare case of multicentric, large, intra-abdominal and retroperitoneal liposarcoma, one of which had caused infection and pressing the right ureter causing hydronephrosis, which was resected by two-stage surgery.

**Case presentation:**

The patient was a 46-year-old man who was referred for abdominal bloating and fatigue. Enhanced computed tomography showed a 23-cm intra-abdominal tumor and a 14.6-cm left retroperitoneal tumor. The intra-abdominal tumor which compressed the right ureter caused right unilateral hydronephrosis and deteriorated the renal function. The intra-abdominal tumor had also formed an intra-abdominal abscess. We performed emergent laparotomy and resected the intra-abdominal tumor. After the recovery of renal function, we resected the residual retroperitoneal tumor. Histopathological examination showed both tumors to be myxoid/round cell type liposarcoma. Considering clinical findings and their location, he was diagnosed with multicentric liposarcoma. He underwent adjuvant chemotherapy and has been alive without any recurrence for 9 months after the operation.

**Conclusions:**

We successfully resected large intra-abdominal and retroperitoneal multicentric myxoid/round cell liposarcomas. A two-stage surgery was a rational choice as it provides time to confirm the recovery of renal function.

## Background

Liposarcoma represents 20–30% of adult soft tissue tumors, which is most frequently seen in the retroperitoneal space in 45% and in the abdominal space only in 5% of cases, but multicentric cases are unknown [1]. It is often detected when it is bigger and heavy, because it rarely presents any symptom during the early period, and many patients who sought clinical help had a chief complaint of abdominal distention or mass [2]. Surgical interventions in liposarcoma differ case by case, according to the relationship with adjacent organs. Herein, we describe a rare case of large, multicentric, intra-abdominal and retroperitoneal liposarcoma, one of which had caused infection and pressed the right ureter causing hydronephrosis, which was resected by a two-stage surgery.

## Case presentation

**Fig. 1:**
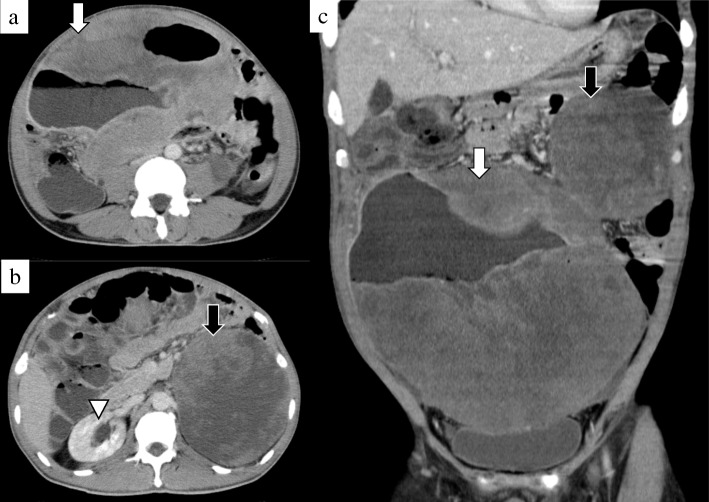
An abdominal enhanced computed tomography scan. **a** An intra-abdominal mass with a diameter of 230 mm (white arrow). The dorsal part of the intra-abdominal tumor contained liquid and air, which indicated abscess formation. **b** A retroperitoneal mass under the left kidney with a diameter of 146 mm (black arrow), intra-abdominal tumor compressed the right ureter, and the right kidney was in hydronephrosis state (white arrowhead). **c** Both tumors had internal inhomogeneity, and the upper part of the intra-abdominal tumor would contain abscess (white arrow, intra-abdominal tumor; black arrow, retroperitoneal tumor)

**Fig. 2:**
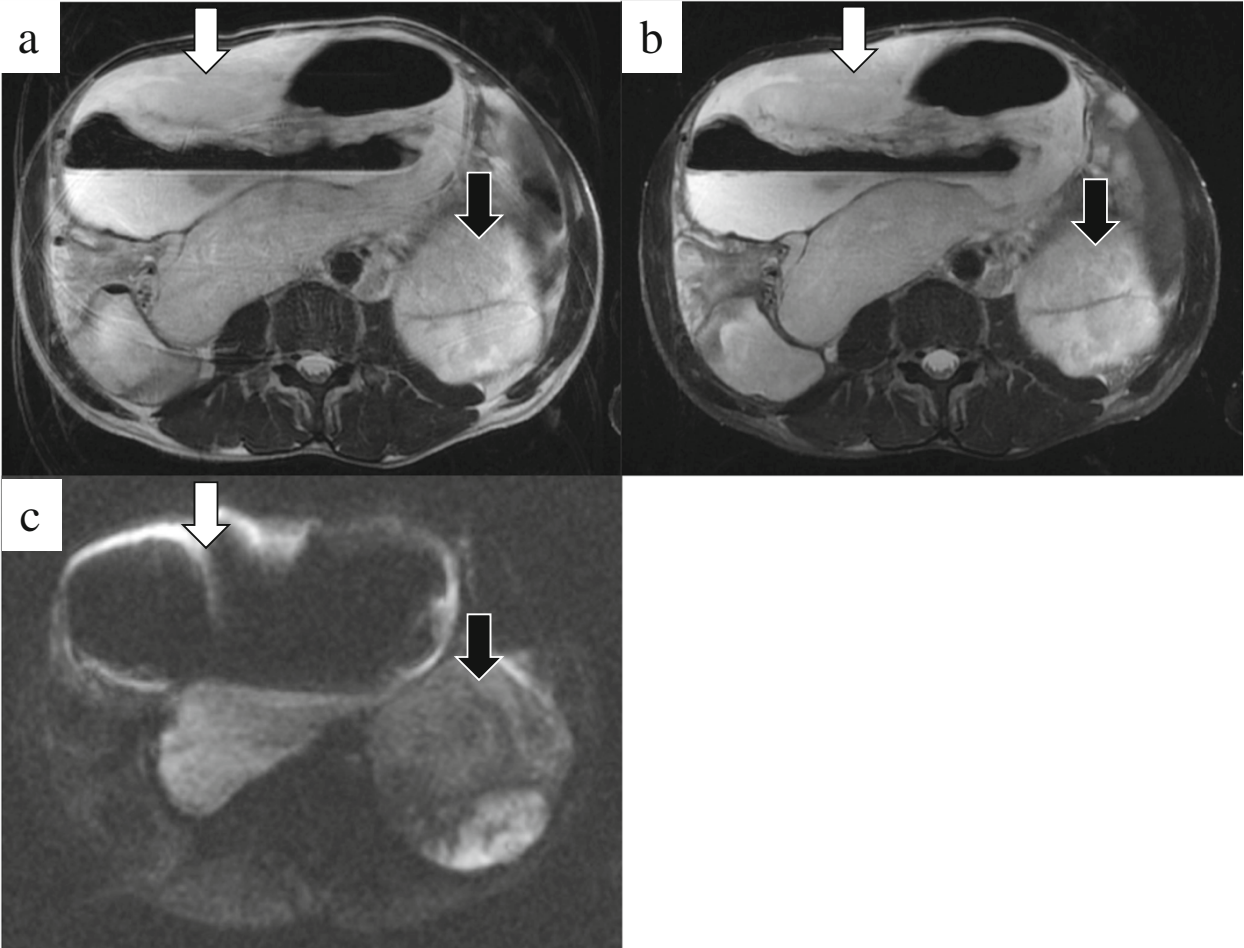
Non-contrast abdominal magnetic resonance image. **a** The solid part of both intra-abdominal (white arrow) and retroperitoneal (black arrow) masses showed inhomogeneously high intensity in T2-weighted imaging (T2WI). **b** Both masses also showed almost the same pattern as T2WI in fat saturation T2WI. **c** Both masses also showed inhomogeneously high intensity in diffusion-weighted imaging

A 46-year-old man complained of heartburn, abdominal distention, and anorexia which persisted for over 2 weeks. He had no significant personal and family history. Body temperature on admission was 39.2 °C. Abdominal physical examination revealed large abdominal masses without tenderness or rebound. Blood examinations showed increased inflammation reaction (white blood cell count, 29,700/μL; C-reactive protein, 33.30 mg/dL). Renal dysfunction was also recognized (Table 1). Abdominal computed tomography revealed two large inhomogeneous masses with a diameter of 230 and 146 mm, respectively. The upper part of the intra-abdominal tumor contained liquid and air, which indicated abscess formation (Fig. 1a–c). As the continuity to the gastrointestinal tract was inexplicit, a mesenchymal tumor was mostly suspected. The tumor compressed the right ureter, which caused right hydronephrosis (Fig. 1b). On the contrary, the left-sided tumor was solid, and it was thought to be derived from the left kidney without left hydronephrosis. Plane abdominal magnetic resonance imaging, T2-weighed imaging (T2WI), and fat saturation T2WI showed mainly high intensity with some low-intensity area (Fig. 2). There is also high intensity in diffusion-weighted imaging and low apparent diffusion coefficient values at solid areas of both masses.

**Table 1:**
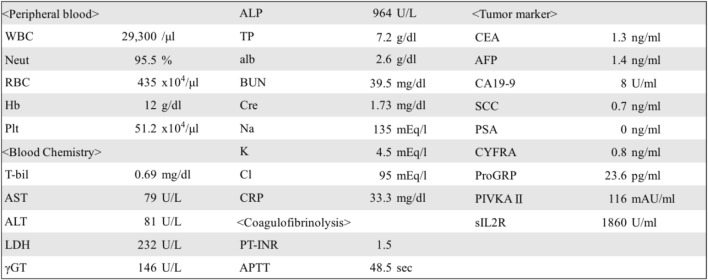
Laboratory test results on admission. Increase in inflammation reaction and renal dysfunction was recognized

*AFP* alpha-fetoprotein, *Alb* albumin, *ALP* alkaline phosphatase, *ALT* alanine aminotransferase, *APTT* activated partial thromboplastin time, *ST* aspartate aminotransferase, *CEA* carcinoembryonic antigen, *Cl* chloride, *Cre* creatinine, *CRP* C-reactive protein, *CYFRA* cytokeratin fragment, *Hb* hemoglobin, *K* potassium, *LDH* lactate dehydrogenase, *NA* sodium, *Neut* neutrophil, *Plt* platelet, *PSA* prostate-specific antigen, *PT-INR* prothrombin time-international normalized ratio, *SCC* squamous cell carcinoma, *T-bil* total bilirubin, *TP* total protein, *WBC* white blood cell, *γGT* gamma-glutamyltransferase

We thought that the intra-abdominal tumor had formed abscess and caused bacterial infection. We decided to resect the right intra-abdominal tumor first to stabilize the general condition from a systemic inflammatory state. After the recovery of renal function, the left retroperitoneal tumor resection was scheduled.

**Fig. 3:**
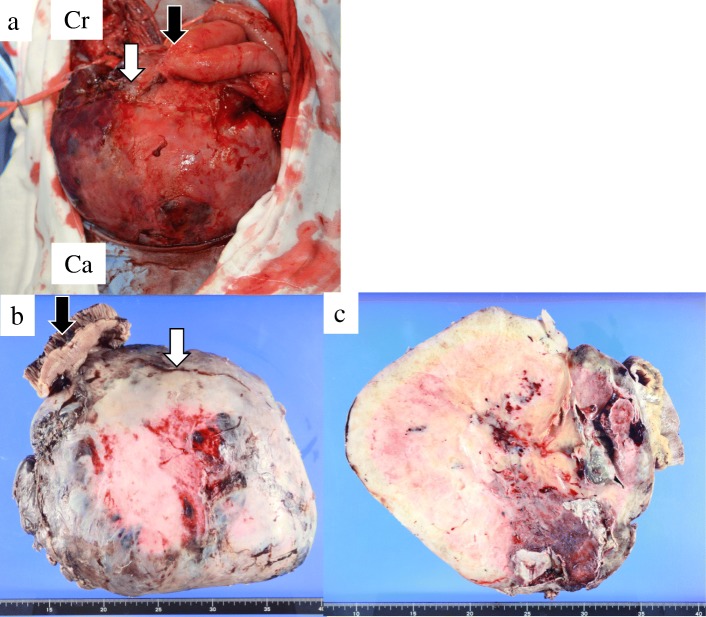
Intraoperative findings of the first surgery and a resected specimen. **a** The intra-abdominal mass (white arrow) invaded the small intestine (black arrow) (Cr, cranial; Ca, caudal). **b** The tumor had a smooth surface. **c** The cut plane looked white and solid inside

**Fig. 4:**
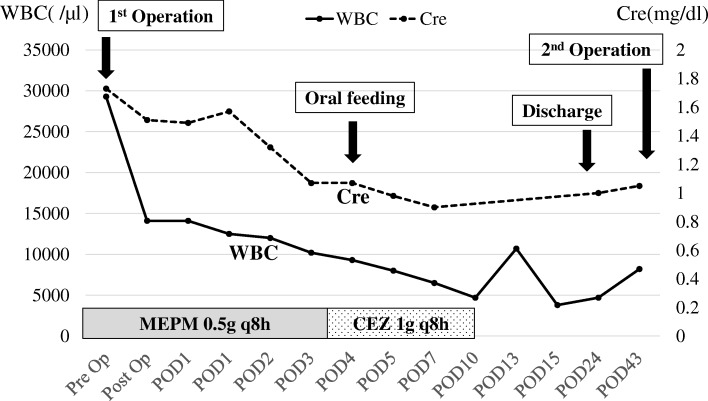
The graph between the first and second operation. The inflammation reaction values and renal function returned to normal after the first operation. On POD 41, the second operation for the retroperitoneal tumor under the left kidney was performed

**Fig. 5:**
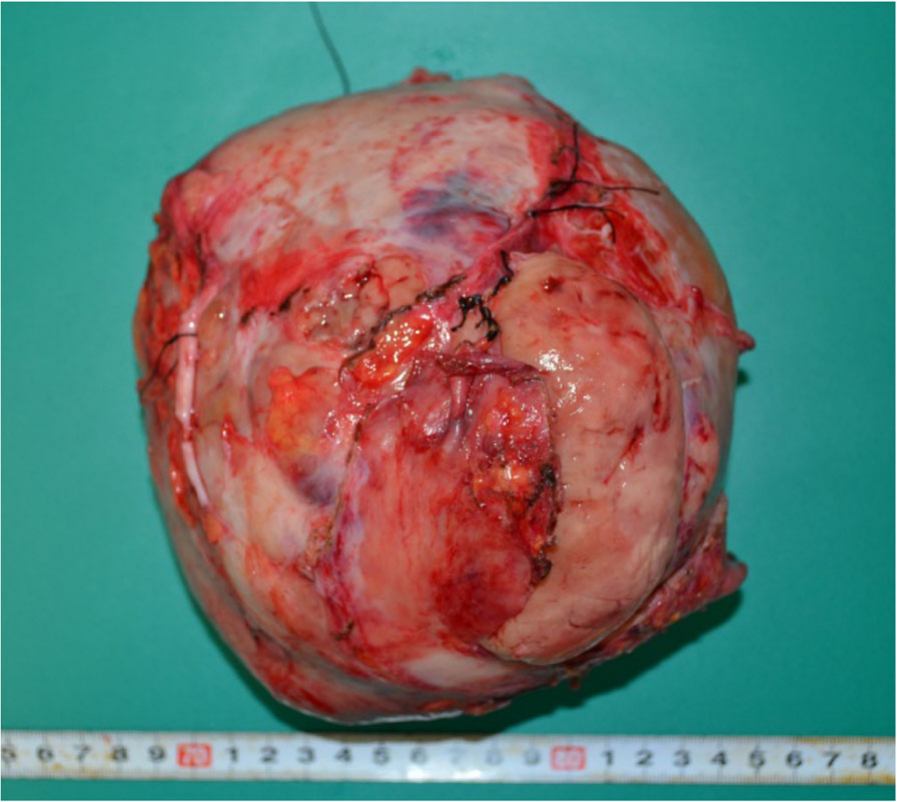
The specimen of the second surgery. The retroperitoneal tumor looked flat and smooth similar to the intra-abdominal tumor

**Fig. 6:**
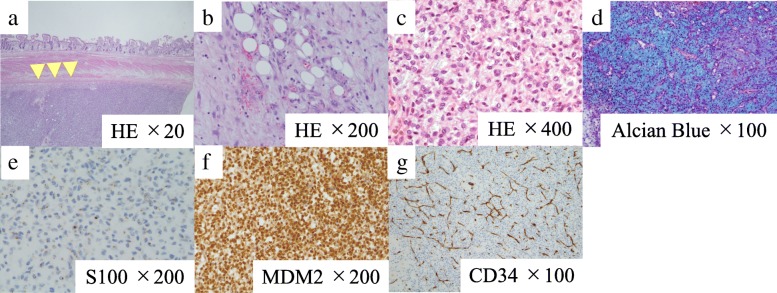
Histopathological examination of the intra-abdominal tumor. **a**–**c** Hematoxylin and eosin-stained sample showed various sizes of spindle-shaped or round-shaped cells in the myxoid parenchyma, surrounded by fibrous capsule, with hyperplasia of the capillary or microvascular vessel, collagen fibers, and adipocyte cells. **a** The small intestine serosa shown above the tumor surface was intact (arrowhead), and no tumor invasion to the small intestine was observed. **d**. Alcian blue staining for the mucinous component was positive. **e**–**g** Immunostaining with S100 and MDM2 was positive and negative with CD34

**Fig. 7:**
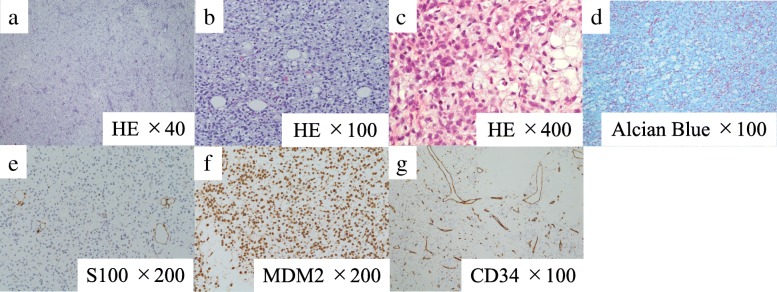
Histopathological examination of the retroperitoneal tumor specimen. **a**, **b** Hematoxylin and eosin-stained sample showed almost the same character with the intra-abdominal tumor, suggesting they had the same differentiation status. **c.** Alcian blue staining for the mucinous component was positive. **d**–**f** Immunostaining with S100 and MDM2 was positive and negative with CD34

In the first surgery, we inserted a ureteral stent to the right ureter and successfully performed resection of the right tumor without invasion to the right ureter. However, the tumor appeared to invade the small intestine, and combined resection was performed (Fig. 3a). The operation time was 274 min, and the total blood loss was 3500 ml. We infused 14 units of red blood cells and 16 units of fresh frozen plasma transfusion. The tumor had smooth surface, 26 × 23 cm in size, and weighed 3.9 kg (Fig. 3b, c). After the first operation, the inflammation reaction values and renal function returned to normal, and he was discharged on postoperative day (POD) 17. On POD 41, the second surgery was performed to remove the retroperitoneal tumor under the left kidney (Fig. 4). Via the same incision as the first surgery, we reached the retroperitoneal tumor transperitoneally. The retroperitoneal tumor was in contact with but not derived from the left kidney, and complete tumor resection was performed without left nephrectomy. The operation time was 224 min, and the total blood loss was 640 ml without any transfusion. Macroscopic findings of the retroperitoneal tumor were similar to those of the right intra-abdominal tumor, and it weighed 1.4 kg (Fig. 5). Histopathological examination of the intra-abdominal tumor on the right side revealed various sizes of spindle-shaped or round-shaped cells in the myxoid parenchyma which were surrounded by fibrous capsule, with hyperplasia of the capillary or microvascular vessel, collagen fibers, and adipocyte cells (Fig. 6a–c). Alcian blue staining for mucinous component was positive in most areas (Fig. 6d). The tumor cells were immunohistochemically positive for S100, MDM2, Rb1, and bcl2, but devoid of CD34, smooth muscle actin, and desmin (Fig. 6e–g). No invasion to the small intestine was seen, and surgical margin was free (Fig. 6a). The characteristics of the retroperitoneal tumor were almost similar to those of the intra-abdominal tumor which suggested that both tumors had the same differentiation status. (Fig. 7a–f). Finally, according to these findings, he was diagnosed with multicentric myxoid and round cell liposarcoma. After the second operation, he was discharged without any complications on POD 14 and received adjuvant triweekly chemotherapy with doxorubicin (30 mg/m^2^) and ifosfamide (2 g/m^2^) for eight courses for 6 months. He has been closely monitored and recurrence-free for 9 months after the first surgery.

Liposarcoma is the most frequent soft-tissue sarcomas. It is usually seen in retroperitoneal (45%), extremities (24%), inguinal, gluteal, and popliteal regions, but it is rarely seen in the intra-abdominal space [3]. Patients with liposarcoma are usually asymptomatic when the tumor is still small; therefore, the tumor is often found after it becomes bigger [2]. Liposarcoma is histologically classified into five categories, namely, well-differentiated (45%), differentiated (20%), pleomorphic (10%), round cell (5%), and myxoid type (rare). Most giant liposarcomas are usually seen as differentiated tumors [4, 5]. Myxoid and round cell tumors share the same characteristic cytogenic abnormalities: the translocation of t(12;16)(q13;p11) leading to the genes’ fusion DDIT3 and FUS with generation of a hybrid protein FUS/DDIT3 [6]. Essentially, round cells are frequently found in myxoid liposarcoma, which is considered to be the marker of poor prognosis when presenting 5% or more of the mass in localized myxoid liposarcoma [7]. The prognosis differs depending on these subtypes as follows: the 5-year survival rate is 95% in well-differentiated type, 92% in mucinous type, 74% in round-cell type, and 59% in pleomorphic type [5].

Liposarcoma is usually a single tumor. When multiple tumors are seen, one of the main problems is to differentiate multicentric lesions from metastases of a single tumor. Multicentric liposarcoma accounts for only 1–2% of all liposarcomas in the USA and Europe [8]. There are no diagnostic criteria, but some features of multicentric liposarcoma are indicated [1]: Primary liposarcomas commonly occur in areas such as the thigh, popliteal fossa, retroperitoneum, peritoneal cavity (including the mesentery and greater omentum), upper extremities, and thoracic serosa [2]. There is no occurrence in sites where metastases are usually found, such as the lungs, liver, and skeletal system [3]. Histopathological type of the tumors is well differentiated or myxoid patterns, which rarely metastasize [4]. There is a long time interval between the occurrence of the first and subsequent tumors [9]. In most cases, it is difficult to differentiate the multicentric liposarcoma from metastatic liposarcoma and there are no criteria. In our case, both liposarcomas had myxoid/round cell types in the common sites and were considered multicentric.

The treatment for liposarcoma has not been established, but en bloc resection is recommended not only for treatment but for pathological diagnosis [5]. Our case developed renal dysfunction due to compression of the right ureter by the intra-abdominal tumor. If we tried to remove both tumors simultaneously, and if the retroperitoneal tumor was derived from the left kidney, composite resection with the kidney was unsafe, as it might affect kidney function. Thus, we decided to first resect the intra-abdominal tumor which caused abdominal abscess.

In this case, multicentric tumors were composed of multiple compartments. Therefore, we thought that his prognosis would be poor and he should undergo chemotherapy in the adjuvant settings. The effectiveness of adjuvant chemotherapy remains unproven, and proper regimen has not been established.

Tumor size (> 20 cm), histological subtypes, and tumor dissemination are considered important prognostic factors [10]. In this case, both tumors were myxoid/round cell types and found in two different compartments. The patient was thought to be at risk for recurrence and received adjuvant chemotherapy, but radiation therapy was not initiated because of the wide range of tumor beds. Myxoid/round cell liposarcoma is a chemosensitive soft tissue tumor, and adjuvant chemotherapy with doxorubicin is recommended [11]. A meta-analysis showed that the effect of the doxorubicin-based chemotherapy improved relapse-free survival, but not overall survival [12]. In this case, he underwent triweekly chemotherapy with doxorubicin (30 mg/m^2^) and ifosfamide (2 g/m^2^) for five cycles, and there has been no recurrence for 9 months. In our case of multicentric myxoid/round cell liposarcoma, en bloc surgical resection and adjuvant chemotherapy were effective.

## Conclusion

We successfully resected large, intra-abdominal, and retroperitoneal multicentric myxoid/round cell liposarcomas. A two-stage surgery was a rational choice as it provides time to confirm the recovery of kidney function.

## Abbreviations

CT: Computed tomography
T2WI: T2-weighed imaging

## References

[1] Roviello F (2007). Giant liposarcoma of the mesentery. Giant liposarcoma of the mesentery. Report of a case. Ann Ital Chir.

[2] Jaques DP, Coit DG, Hajdu SI, Brennan MF (1990). Management of primary and recurrent soft-tissue sarcoma of the retroperitoneum. Ann Surg.

[3] McKinley SK, Abreu N, Patalas E, Chang A (2013). Large retroperitoneal liposarcoma diagnosed upon radiological evaluation of mild right-sided inguinal hernia. Case Rep Radiol.

[4] Eltweri AM, Gravante G, Read-Jones SL, Rai S, Bowrey DJ, Haynes IG (2013). A case of recurrent mesocolon myxoid liposarcoma and review of the literature. Case Rep Oncol Med.

[5] Sharma M, Mannan R, Bhasin TS, Manjari M, Punj R (2013). Giant inflammatory variant of well differentiated liposarcoma: a case report of a rare entity. J Clin Diagn Res.

[6] Göransson M, Andersson MK, Forni C, Ståhlberg A, Andersson C, Olofsson A, Mantovani R, Aman P (2009). The myxoid liposarcoma FUS-DDIT3 fusion oncoprotein deregulates NF-kappaB target genes by interaction with NFKBIZ. Oncogene.

[7] Fernández-Aceñero MJ, López-Criado P, López-Franco M, Meizoso T, Calvo C (2007). Multicentric myxoid liposarcoma: report of two cases. World J Surg Oncol.

[8] Pack GT, Pierson JC (1954). Liposarcoma: a study of 105 cases. Surgery.

[9] Seenu V, Kriplani AK, Shukla NK, Raina V, Thakur K, Kapur BM (1995). Multicentric liposarcoma: report of two cases. Surg Today.

[10] Duman K, Girgin M, Artasc G (2016). A case report: giant intra-abdominal liposarcoma presenting acute renal failure. Ann Med Surg (Lond).

[11] Grimer R, Judson I, Peake D, Seddon B (2010). Guidelines for the management of soft tissue sarcomas. Sarcoma.

[12] O’Connor JM, Chacón M, Petracci FE, Chacón RD (2008). Adjuvant chemotherapy in soft tissue sarcoma (STS): a meta-analysis of published data. J Clin Oncol.

